# Translation and validation of the OHIP-14 Oral Health Impact Profile into the Quechua language of Peru

**DOI:** 10.1590/1807-3107bor-2025.vol39.098

**Published:** 2025-10-20

**Authors:** Jhair Alexander LEON-RODRIGUEZ, María ESPINOZA-SALCEDO, Yovana Melisza GUTIERREZ-POLANCO, Rudyard Wilhelm AQUEPUCHO-BELLOTA, Jherson David LEON-RODRIGUEZ, Juan Luis LEON-PLASENCIA

**Affiliations:** (a) Antenor Orrego Private University, Postgraduate School, Trujillo, Peru.; (b) Andean University of Cusco, School of Nursing, Cusco, Peru.; (c) Northern Private University, Postgraduate School, Trujillo, Peru.

**Keywords:** Oral Health, Translations, Validation Study, Peru

## Abstract

The aim of this study was to translate and validate the 14-item Oral Health Impact Profile (OHIP-14) into the Collao variant of the Quechua language for the population of southern Peru. A cross-sectional instrumental study was carried out with 288 participants of both sexes aged between 18 and 65 years (M = 37.53 years; SD = 10.38) who were residents of the department of Cusco, Peru. A convenience sampling method was used. The cultural adaptation process included initial translation, back-translation, review by a committee, focus group evaluation for comprehension and clarity of the items, and a pilot test to ensure the cultural adequacy of the questionnaire. Content validity was evaluated by three evaluators using Aiken’s V coefficient. The internal structure was assessed through confirmatory factor analysis (CFA), which tests a three-factor model. The fit indices demonstrated an acceptable model fit, with χ^2^ = 156.25 (df = 74), RMSEA = 0.062 (90%CI: 0.049–0.076), CFI = 0.999, and TLI = 0.999. The standardized factor loadings exceeded 0.70 for all the items. The corrected item–total correlations indicated strong internal consistency. Reliability was assessed with Cronbach’s alpha, which is a high value across all dimensions. The evaluators’ assessment demonstrated a high level of agreement for all the items, with Aiken’s V coefficient above 0.70. This finding demonstrates that the OHIP-14 is a valid and reliable instrument for the Quechua-speaking population of the Collao variant in Cusco. The study received approval from an ethics committee and adhered to the ethical principles of the Declaration of Helsinki.

## Introduction

The impact of oral health on quality of life is a topic of interest within dentistry.^1^ Evaluating the impact of oral conditions on quality of life is a fundamental component in assessing oral health needs;^2^ this is because clinical indicators, by themselves, are insufficient to describe patients’ satisfaction, their symptoms, or their ability to perform daily activities. In particular, oral health-related quality of life (OHRQoL) indicators are widely applied in both cross-sectional and longitudinal studies to assess how oral health affects overall well-being and functionality.^3-6^

The Oral Health Impact Profile (OHIP-14) questionnaire is composed of 14 items designed to assess self-reported functional limitations, discomfort, and disability associated with oral conditions.^7^ This questionnaire originates from a longer version with 49 items,^8^ based on a theoretical model developed by the World Health Organization and adapted to address oral health problems by Locker.^9^ According to this model, the consequences of oral diseases are hierarchically linked from a biological level (impairment) to a behavioral level (functional limitation, discomfort and disability), and, finally, to the social level (handicap). Despite its brevity, the OHIP-14 has been shown to be reliable,^7^ to be sensitive to changes,^9,10^ and to have adequate cross-cultural coherence.^11^

The OHIP-14 is among the most widely used quality-of-life indicators related to oral health used internationally, and it is accessible in various languages, including Chinese, Finnish, French, German, Japanese, Malay, Portuguese, Sinhalese, Somali, and Swedish.^5^

Although a version in Peruvian Spanish has recently been validated through an exhaustive study involving a large sample of Peruvians,^12^ it fails to address the linguistic needs of indigenous communities, which continue to face persistent challenges, including language barriers. The translation of the OHIP-14 into the Quechua Collao was carried out from this validated Peruvian Spanish version, ensuring fidelity in the adaptations made for the Peruvian context.

Even when efforts were made to bring dentistry to rural areas through teledentistry, language was a significant barrier, as in the case of the Primary Care Center of Pichanaki, located in the central Peruvian jungle, where teleodontology was implemented to improve access to dental services amid geographical and logistical challenges; this highlights the vital importance of overcoming language barriers to ensure effective and equitable access to oral health services.^13^

Peru stands out for its rich cultural diversity, being a multicultural country inhabited since ancient times by diverse groups with different traditions and perspectives on life and well-being.^14^ Among these groups, there is a community that communicates through the native Quechua language, presenting variants in the Amazon and northern, central, and southern regions. The latter is subdivided into two variants, Quechua Chanka and Quechua Collao, which are used in the districts of Apurímac, Cusco, Puno, Arequipa, and Moquegua.^15^

These language barriers not only hinder the collection of accurate data for research but also limit these communities’ access to quality oral health services.^16^ The historical challenge of conducting studies requiring communication in native languages has prevented an adequate assessment of the specific needs of indigenous communities, perpetuating inequalities in care. Indigenous populations in central and southern Peru experience significant disparities in oral health, and language barriers significantly contribute to the persistence of these disparities.^17^ The lack of effective communication prevents professionals from understanding the specific needs of these populations and, in turn, hinders patients’ understanding of the recommended treatments or interventions.^18^

Given the urgency of addressing the needs of indigenous communities residing in rural areas of southern Peru, where the impact of public health disparities tends to be more pronounced, it is essential to have culturally adapted oral health impact profile assessment tools. To partially address this need, a previous study translated the OHIP-14 instrument into Peruvian Spanish and demonstrated its validity and reliability.^12^ However, this version fell short of meeting the linguistic needs of indigenous Quechua-speaking populations, particularly those using the Collao variant.

Cusco is a city with approximately 1,205,527 inhabitants, of whom 609,655 are speak Quechua of the Collao variant. The districts that constitute the largest proportion of the Quechua-speaking population are Checca in the province of Canas and Ccorca in the province of Cusco.^19^

A cross-sectional descriptive study conducted in 2018 in the native communities of Potsoteni, Boca Sanibeni, and Unión Puerto Ashaninka, located in Central Peruvian Jungle Province, Junín Department, Peru, with a sample of 169 adults, revealed that 68.63% of the population presented with malocclusions and that 100% suffered from dental caries. Additionally, 68% of the patients experienced clinical complications resulting from untreated dental caries. The average score for the Simplified Oral Hygiene Index (IHO-S) was 5.02, indicating poor oral hygiene conditions in most cases. Furthermore, 21.3% of the population require fixed upper prostheses, whereas 17.4% need partial removable prostheses for the lower jaw, highlighting that edentulism remains a persistent oral health problem. On this basis, addressing linguistic barriers and cultural diversity within these communities must be a priority for the public health system to ensure a more equitable assessment and provision of oral health care.^20^

This study was conducted to translate and validate the Oral Health Impact Profile (OHIP-14) into Collao Quechua, addressing the linguistic and cultural needs of indigenous communities in southern Peru.

## Methods

### Study design

This was a cross-sectional and instrumental study for the adaptation and validation of the Oral Health Impact Profile (OHIP-14) for the Quechua-speaking population (Collao variant) of Cusco, Peru.

Nonprobability convenience sampling was used to determine the sample. The inclusion criteria were being of legal age, providing informed consent and communicating in Quechua (understands, reads, writes). The exclusion criterion was understanding Quechua. The sample included 288 Quechua speakers, of whom 143 were men (49.7%) and 145 were women (50.3%), with a mean age of 37.53 ± 10.38 years. The most commonly reported marital status was single, with 105 participants (36.5%) (Table 1).

**Table 1:** Characteristics of Cusco’s Collao Quechua speakers.

	M	SD
Age	37,53	± 10.38
	N	%
Sex		
Male	143	49,7
Female	145	50,3
Marital status		
Maried	89	30,9
Single	105	36,5
Divorced	94	32,6

M = Mean; SD = Standard deviation.

### Procedure

The study was conducted from July to November 2023 under the supervision of Dr. Jhair Alexander Leon Rodriguez, who rigorously performed and supervised each phase of the process, consisting of two stages: validation and translation of the Oral Health Impact Profile (OHIP-14) from Peruvian Spanish to the Collao variant of Quechua spoken in Cusco, Peru. The items of the OHIP-14 were translated into Collao Quechua from a validated version and adapted into Peruvian Spanish by Becerra and Condori,^12^ as described in their study on the adaptation and validation of the OHIP-14 in a Peruvian population. The translation was conducted with the explicit authorization of the original author, obtained via email correspondence, ensuring compliance with copyright regulations and proper use of the instrument.

The translation process was conducted according to the standards detailed by Sousa and Rojjanasrirat,^21^ beginning with an initial translation by two translators, followed by a back-translation performed by another independent translator who had no access to the original document, ensuring the objectivity of the process.

### Initial translation

Two professional translators were invited to translate the Peruvian Spanish version of the Oral Health Impact Profile (OHIP-14) into the Collao variant of Quechua spoken in Cusco. The translators, who are proficient in both Collao Quechua and Peruvian Spanish, independently translated the questionnaire items, taking into account the cultural and linguistic particularities of both languages. Both subsequently met with a Peruvian dental surgeon specializing in pediatric dentistry whose mother tongue was Collao Quechua who had experience in evaluating oral health in children and with a Peruvian dental surgeon expert in instrument translation. The four formed a review committee to consolidate the work. After a joint agreement, the consolidated version of the OHIP-14 was presented in Collao Quechua.

### Back translation.

The consolidated version of the OHIP-14 in Collao Quechua was sent to two additional translators, who, without prior knowledge of the study, independently translated the items; this was done to ensure objectivity and to verify the consistency and accuracy of the translated version. After completing the back translation, they met with a Collao Quechua-speaking dental surgeon and a researcher registered in Peru’s National Registry of Science, Technology, and Technological Innovation (RENACYT), who had a doctorate in stomatology, to verify the quality of the process and analyze the similarities and differences between the Peruvian Spanish version and the version translated into the Collao variant of Quechua. After verifying that the translated items met the measurement requirements for the constructs of the original version, the final version was approved.

### Validation of the questionnaire

The validation of the questionnaire consisted of a review by experts. Initially, three dental surgeons with master’s degrees participated in the expert review, two of whom had at least 3 years of work experience in the health field and one who experience in university teaching. The review sheet included a space in which the expert could make qualitative suggestions for each item concerning the relevance, representativeness, and clarity of the words in the Collao Quechua variant. A quantitative component was also included in which experts assigned scores to each item. Content validity was calculated using Aiken’s V for the items’ relevance, representativeness, and clarity, with significant values above 0.70. After the experts’ recommendations were incorporated, a preliminary Collao Quechua version was produced (Table 2), which was subsequently evaluated by a focus group.

**Table 2:** Final translation of the OHIP-14 into Cusco’s Collao Quechua

No	Items of the Spanish version	Items in Collao Quechua
1	¿Ha tenido dificultad para pronunciar palabras, por problemas en dientes o boca?	¿Sasachu karan rimanaykipaq kiruyki otaq simiykipi sasachakuykuna?
2	¿El sabor de sus alimentos ha empeorado por problemas en dientes o boca?	¿Millay mikhusqaykita aswan millayta tukuchin sasachakuy kuna kiruyki otaq simiykipi?
3	¿Ha sentido dolor en dientes o boca?	¿kiru nanaywuan otaq simi nanaiwanchu karanki?
4	¿Ha presentado molestia al comer?	¿Sasachakuwan chu mikhuqtiki karanki?
5	¿Le preocupan los problemas de sus dientes o boca?	¿Llakisqachu karanki kiruyki otaq simikipi sasachakuykunawan?
6	¿Se ha sentido nervioso o estresado, por problemas en dientes o boca?	¿Mancharisqa otaq llakisqachu karanki kiruyki otaq simiykipi sasachukuyqwan?
7	¿Ha tenido que cambiar sus alimentos, por problemas en dientes o boca?	¿Mikhunayki rantinninta tariran kichu kiruyki otaq simaykipi sasachakuykunawan?
8	¿Ha tenido que interrumpir sus alimentos, por problemas en dientes o boca?	¿Manan mikhu naysunkichu kiruyki otaq simiykipi sasachakuy kunawan?
9	¿Ha encontrado dificultad para descansar, por problemas en dientes o boca?	¿Sasa puñuytachu tarikuranki kiruyki otaq simiykipi sasakuykunawan?
10	¿Se ha sentido avergonzado por problemas con sus dientes o boca?	¿P’enqarisqachu karanki kiruykiwan otaq simiykipi sasachakuynawan?
11	¿Ha estado irritable debido a problemas en dientes o boca?	¿kiruykiwan otaq simiykiwan sasachakuykuna kasqanraykuchu piñakurqanki?
12	¿Ha tenido dificultad para realizar sus actividades diarias por problemas en dientes o boca?	¿Sasachakuykuran kichu sapa p’unchay ruwaqaykikunawan kiruykiwan otaq simiykipi sasachakuykunawan?
13	¿Ha sentido que la vida en general ha sido menos agradable por problemas en dientes o boca?	¿Yuyaynikiwan kausaynikipi millaychukaran kiruykiwan otaq simiykipi sasachaykunawan?
14	¿Las molestias en dientes o boca, le han impedido hacer tu vida normal?	¿Kiruykikunapu otaq simiykipi sasachaykuna mana allin kausay tachu tarikuranki?

### Focus group

A dental surgeon researcher, who was an expert in qualitative data collection methods, organized a meeting with 11 Collao Quechua speakers (6 men and 5 women). They were all over 18 years old and had finished high school, so they knew how to read and write in Collao Quechua and Peruvian Spanish.

Initially, the participants were asked to read the written version of the OHIP-14 in Collao Quechua and complete it. After administration, the moderating dental surgeon facilitated a discussion on the clarity and comprehension of the items. No modifications were deemed necessary, so the preliminary version was confirmed as the final version of the Oral Health Impact Profile (OHIP-14) in Collao Quechua (Table 2).

### Application of the validated questionnaire

After the OHIP-14 was validated by expert judges and a focus group, a virtual form was designed via Google Forms. Informed consent, sociodemographic data, and questionnaire statements translated into Collao Quechua were included. Participation in the questionnaire was entirely voluntary and strictly anonymous. The information collected was used exclusively for research purposes. While the survey was administered in Collao Quechua, it was limited to participants who could fluently read and understand the language; in the study region, all of the eligible Quechua speakers were functionally bilingual owing to the widespread use of Spanish.

### Instrument

The Oral Health Impact Profile (OHIP-14) was validated in Peruvian Spanish by Becerra and Condori^12^ and then translated and validated into Collao Quechua; it consists of 14 items evaluating 7 dimensions (functional limitations, physical pain, psychological discomfort, physical disability, psychological disability, social disability and handicap). The OHIP-14 allows the quantification of the product of oral health with respect to the quality of life of patients and consists of a scale of 5 options (0 = Never, 1 = Almost never, 2 = Occasionally, 3 = Frequently, 4 = Always).

### Statistical analysis

For content validity, quantitative data for the items were gathered with evaluation by three dental surgeons with master’s degrees. Aiken’s V coefficient was calculated to evaluate the content validity of the items, with a focus on their relevance, representativeness, and clarity. Items with values equal to or greater than 0.70 were retained in the final version of the instrument.^22^

A descriptive statistical analysis of the items (mean, standard deviation, skewness, and kurtosis) was subsequently performed. Values outside the range of -2 to +2 were considered indicative of non-normal distribution for skewness and kurtosis.^23^

Confirmatory factor analysis (CFA) was conducted to establish the construct validity of the instrument through structural equation modeling using R Studio and the lavaan package.^24^ The weighted least squares mean and variance adjusted (WLSMV) estimator, which is suitable for ordinal response formats, was applied. Two theoretical models were evaluated: a single-factor model and a three-factor model. In the single-factor model, all of the items are grouped under one dimension, whereas in the three-factor model, the items are distributed across three dimensions: psychosocial impact, functional limitation, and pain-discomfort.

Goodness-of-fit indices, including the adjusted goodness-of-fit index (AGFI), goodness-of-fit index (GFI), Tucker–Lewis index (TLI), comparative fit index (CFI), and root mean square error of approximation (RMSEA), were considered on the basis of the criteria established by Hu and Bentler.^24^ For the RMSEA, a 90% confidence interval was applied. These indices were used to evaluate the theoretical alignment and internal consistency of the instrument with its proposed multidimensional structure. The analysis was conducted for both models: the single-factor model and the three-factor model.

The construct reliability was assessed with Cronbach’s alpha coefficient, with a 95% confidence interval.^26^ Descriptive and reliability analyses were conducted using SPSS version 26.0.

### Ethical considerations

This study was approved by the Research Ethics Committee of Trujillo Public University with resolution No. P.I.B. EST.–011-2023, and all of the ethical principles of the Declaration of Helsinki for research involving human subjects were followed.

## Results

This section presents the results of the content analysis, focusing on the evaluation of the relevance, representativeness, and clarity of the OHIP-14 items. All the items received a high level of agreement, with values of Aiken’s V ≥ 0.70 (Table 3), confirming that the items met the criteria for content adequacy.

**Table 3:** Aiken’s V for the assessment of relevance, representativeness, and clarity of the items in the OHIP-14

Items	Relevance (n=3)				Representativeness (n=3)				Clarity (n=3)			
	Mean	SD	V	95% CI	Mean	SD	V	95% CI	Mean	SD	V	95% CI
Item 1	4.33	0.58	0.83	0.55-0.95	4.67	0.58	0.92	0.65-0.99	4.67	0.58	0.92	0.65-0.99
Item 2	4.67	0.58	0.92	0.65-0.99	4.67	0.58	0.92	0.65-0.99	4.67	0.58	0.92	0.65-0.99
Item 3	4.33	0.58	0.83	0.55-0.95	4.00	0.00	0.75	0.47-0.91	5.00	0.00	1.00	0.76-1.00
Item 4	5.00	0.00	1.00	0.76-1.00	4.67	0.58	0.92	0.65-0.99	4.67	0.58	0.92	0.65-0.99
Item 5	4.33	0.58	0.83	0.55-0.95	4.33	0.58	0.83	0.55-0.95	5.00	0.00	1.00	0.76-1.00
Item 6	4.67	0.58	0.92	0.65-0.99	4.33	0.58	0.83	0.55-0.95	4.33	0.58	0.83	0.55-0.95
Item 7	5.00	0.00	1.00	0.76-1.00	5.00	0.00	1.00	0.76-1.00	4.67	0.58	0.92	0.65-0.99
Item 8	5.00	0.00	1.00	0.76-1.00	4.67	0.58	0.92	0.65-0.99	4.67	0.58	0.92	0.65-0.99
Item 9	4.33	0.58	0.83	0.55-0.95	4.00	0.00	0.75	0.47-0.91	5.00	0.00	1.00	0.76-1.00
Item 10	5.00	0.00	1.00	0.76-1.00	5.00	0.00	1.00	0.76-1.00	5.00	0.00	1.00	0.76-1.00
Item 11	4.33	0.58	0.83	0.55-0.95	4.33	0.58	0.83	0.55-0.95	4.67	0.58	0.92	0.65-0.99
Item 12	4.33	0.58	0.83	0.55-0.95	4.67	0.58	0.92	0.65-0.99	4.67	0.58	0.92	0.65-0.99
Item 13	5.00	0.00	1.00	0.76-1.00	4.67	0.58	0.92	0.65-0.99	5.00	0.00	1.00	0.76-1.00
Item 14	4.67	0.58	0.92	0.65-0.99	4.33	0.58	0.83	0.55-0.95	4.33	0.58	0.83	0.55-0.95

SD = Standard deviation, V = Aiken’s V.

### Preliminary analysis of the items

The descriptive statistics for the 14 items of the OHIP-14, including the mean, standard deviation, skewness, and kurtosis, were analyzed. Item 10 had the highest mean score (M = 4.087), and Item 14 had the highest variability (SD = 0.780). The skewness and kurtosis values for all the items remained within the acceptable range of ± 2. The corrected item–total correlations are also displayed. All the items presented correlation values above 0.60, reflecting a consistent contribution to the total score. The item-deleted Cronbach’s α values were consistently greater than 0.80 (Table 4).

**Table 4:** Preliminary analysis of the items in the recovery experiences questionnaire.

Variable	M	SD	A	K	H	r itc	α
Item 1	4.031	0.744	-0.050	-1.184	0.688	0.816	0.970
Item 2	3.938	0.803	0.073	-1.348	0.707	0.829	0.969
Item 3	3.958	0.791	0.074	-1.396	0.716	0.833	0.969
Item 4	3.944	0.799	0.100	-1.425	0.694	0.820	0.970
Item 5	3.924	0.810	0.101	-1.378	0.723	0.838	0.969
Item 6	3.944	0.799	0.100	-1.425	0.719	0.835	0.969
Item 7	3.910	0.817	0.168	-1.483	0.733	0.843	0.969
Item 8	3.931	0.806	0.127	-1.451	0.723	0.837	0.969
Item 9	3.962	0.789	0.025	-1.286	0.687	0.816	0.970
Item 10	3.965	0.787	0.061	-1.380	0.687	0.816	0.970
Item 11	3.931	0.806	0.087	-1.364	0.716	0.834	0.969
Item 12	3.948	0.797	0.052	-1.323	0.712	0.830	0.969
Item 13	3.920	0.812	0.147	-1.468	0.723	0.838	0.969
Item 14	3.962	0.789	0.025	-1.286	0.700	0.824	0.969

Note: M = Mean; SD = Standard deviation; As = Skewness coefficient; K = Kurtosis coefficient; H = Commonality; r itc = Correlation item-corrected test; α = Cronbach alpha.

### Confirmatory factor analysis

Two models were tested: a single-factor model and a three-factor model. Both models demonstrated acceptable fit indices. For the single-factor model, the fit indices were χ^2^ = 158.68 (df = 77), RMSEA = 0.061 (90%CI: 0.047–0.074), CFI = 0.999, TLI = 0.999, and SRMR = 0.036. The three-factor model presented χ^2^ = 156.25 (df = 74), RMSEA = 0.062 (90%CI: 0.049–0.076), CFI = 0.999, TLI = 0.999, and SRMR = 0.036 (Table 5).

**Table 5:** Model fit indices assessed through Confirmatory Factor Analysis (CFA) of the study’s instrument

Model	χ^2^	df	GFI	CFI	TLI	RMSEA (90% CI)	RMR
One-factor model	158.675	77	0.998	0.999	0.999	0.061 (0.047-0.074)	0.032
Three-factor model	156.249	74	0.998	0.999	0.999	0.062 (0.049-0.076)	0.031

Note: The models were assessed using the DWLS (Diagonally Weighted Least Squares) estimator.

The standardized factor loadings for the three-factor model are presented in Table 6. All loadings exceeded 0.70, with the highest values observed for Items P11 (0.999) and P3 (1.009). The error covariances among the items in the three-factor model were evaluated (Table 7). The highest residual covariance was -0.079 between Items P12 and P13, whereas the other covariances ranged from -0.096 to 0.060.

**Table 6:** Standardized factor loadings for the three-factor model of the OHIP-14.

Items	Dimension	Standardized Factor Loading
Item 1	Functional Limitation	1.000
Item 2	Functional Limitation	1.019
Item 3	Pain-Discomfort	1.000
Item 4	Pain-Discomfort	0.976
Item 5	Psychosocial Impacts	1.000
Item 6	Psychosocial Impacts	1.001
Item 7	Pain-Discomfort	1.009
Item 8	Pain-Discomfort	1.006
Item 9	Psychosocial Impacts	0.978
Item 10	Psychosocial Impacts	0.964
Item 11	Psychosocial Impacts	0.999
Item 12	Psychosocial Impacts	0.986
Item 13	Psychosocial Impacts	0.995
Item 14	Psychosocial Impacts	0.971

**Table 7:** Error covariances among items in the three-factor model of the OHIP-14

Item	5	6	9	10	11	12	13	14	3	4	7	8	1	2
5	0,000	0,009	0,002	-0,096	0,022	0,014	-0,018	0,019	-0,087	0,020	-0,003	0,018	0,047	-0,006
6	0,009	0,000	0,005	-0,005	-0,024	0,027	-0,022	-0,068	0,000	0,011	-0,013	-0,024	0,048	0,007
9	0,002	0,005	0,000	0,049	0,007	0,013	-0,001	0,012	-0,076	0,025	0,015	0,009	-0,094	0,016
10	-0,096	-0,005	0,049	0,000	0,016	-0,068	0,050	-0,047	0,060	0,032	0,005	-0,002	0,003	-0,055
11	0,022	-0,024	0,007	0,016	0,000	0,049	-0,012	0,040	-0,008	-0,065	-0,020	0,023	-0,100	-0,017
12	0,014	0,027	0,013	-0,068	0,049	0,000	-0,079	0,030	0,010	-0,092	0,026	0,031	-0,056	0,006
13	-0,018	-0,022	-0,001	0,050	-0,012	-0,079	0,000	-0,068	0,032	0,048	0,003	-0,013	0,016	0,013
14	0,019	-0,068	0,012	-0,047	0,040	0,030	-0,068	0,000	0,021	-0,037	0,016	-0,059	0,044	0,044
3	-0,087	0,000	-0,076	0,060	-0,008	0,010	0,032	0,021	0,000	-0,019	-0,003	-0,014	0,005	0,029
4	0,020	0,011	0,025	0,032	-0,065	-0,092	0,048	-0,037	-0,019	0,000	0,019	0,003	0,016	0,018
7	-0,003	-0,013	0,015	0,005	-0,020	0,026	0,003	0,016	-0,003	0,019	0,000	0,005	0,021	-0,090
8	0,018	-0,024	0,009	-0,002	0,023	0,031	-0,013	-0,059	-0,014	0,003	0,005	0,000	-0,017	0,006
1	0,047	0,048	-0,094	0,003	-0,100	-0,056	0,016	0,044	0,005	0,016	0,021	-0,017	0,000	0,000
2	-0,006	0,007	0,016	-0,055	-0,017	0,006	0,013	0,044	0,029	0,018	-0,090	0,006	0,000	0,000

*Fnc* refers to Functional Limitation; *Pan* refers to Pain-Discomfort; *Phy* refers to Psychosocial Impacts.

The results of the confirmatory factor analysis support the three-factor model of the OHIP-14 scale, which includes the dimensions of functional limitations, pain-discomfort, and psychosocial impacts (Figure).

**Figure:**
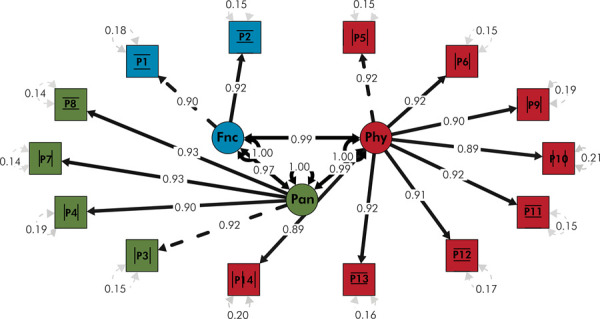
Three-Factor Model of the OHIP-14 Scale – Quechua Collao Version (Functional Limitation, Pain-Discomfort, Psychosocial Impacts)

## Discussion

There is a growing interest in measuring oral health-related quality of life in intercultural settings; this demonstrates the importance of having valid and reliable instruments that can be used in different geographical and cultural contexts, making it essential to translate these instruments into native languages.

This study addresses the translation and cross-cultural adaptation of the OHIP-14 into Collao Quechua, which is a language spoken by indigenous communities in southern Peru. As noted, the process adhered to the guidelines proposed by Sousa and Rojjanasrirat^21^ and Beaton et al.,^27^ which provided complementary frameworks for cross-cultural adaptation. The former emphasized a clear, systematic approach to translation and validation in health care research, whereas the latter outlined specific steps for adapting self-report measures across cultures. Oral health-related quality-of-life instruments are multidimensional.^28^ The dimensions provide an informative way to profile important oral health data, and the OHIP-14 divides the questionnaire into seven dimensions based on expert opinions and a conceptual model of oral health.^8^

This is the first cross-cultural adaptation of the OHIP-14 into the Collao Quechua language, a native language of South America. After favorable evidence for clarity, representativeness, and relevance of the translated items was obtained through dental surgeons’ evaluations, the analysis of psychometric properties demonstrated that the indicators were acceptable.

The validity of the internal structure determined through confirmatory factor analysis revealed appropriate fit indices for the multidimensional model to measure the Oral Health Impact Profile. These results align with previous cross-cultural adaptations. The Danish version of the OHIP-14 demonstrated that the instrument maintains good face and content validity, internal consistency, and reliability when adapted to a different linguistic and cultural context.^29^ Similarly, the Persian version of the OHIP-14 confirmed the instrument’s ability to reliably measure oral health-related quality of life, highlighting its robust convergent and discriminative validity.^30^ Furthermore, the German version of the OHIP-14 validated the multidimensionality and internal consistency of the instrument, ensuring its applicability across diverse populations.^31^ These studies confirmed the reliability and validity of the instrument when adapted and validated in diverse languages and populations. This is consistent with the original version. Based on these findings and their consistency with the results, the multidimensional structure of the OHIP-14 translated into the Quechua Collao has reasonable empirical support.

In this study, both the single-factor and three-factor models showed acceptable fit indices. However, the three-factor model demonstrated marginally better fit. The standardized factor loadings were all above 0.70, providing evidence for the instrument’s internal structure. Furthermore, the internal consistency observed (Cronbach’s α = 0.97) indicates excellent reliability and strong coherence among the items in measuring the intended construct. The high Cronbach’s alpha value obtained is justifiable based on the length of the questionnaire, as longer instruments such as the OHIP-14, which contains 14 items, often exhibit elevated alpha values; this does not necessarily indicate redundancy but rather reflects a well-defined construct and strong internal consistency. This level of reliability is consistent with prior validations of the OHIP-14 in Malay,^32^ Danish,^29^ and Spanish^31^ populations. Taken together, these findings support the conclusion that the OHIP-14 translated into the Quechua Collao is a valid, reliable, and multidimensional instrument for assessing oral health-related quality of life among indigenous Quechua-speaking populations in Peru.

There are certain limitations that we find necessary to report. First, the sample size analyzed was nonprobabilistic, limiting the generalizability of the results, as all participants were from the Cusco region in Peru. Future studies should evaluate the psychometric properties of the OHIP-14 translated into the Quechua Collao in different geographical areas of indigenous communities in southern Peru, such as the departments of Moquegua, Arequipa, Puno, and Apurímac.

Second, only internal consistency and content validity were assessed, so it is recommended that future studies complement these findings with external criteria, such as a stomatological diagnosis, to gather more evidence on validity in relation to other variables. Finally, reliability was not analyzed via the test–retest technique, so conclusions about its measurement stability cannot be drawn. These aspects should be included in future research agendas.

Despite these aforementioned limitations, the results are significant. The practical relevance lies in providing a validated instrument in Quechua, specifically the Collao variant, with evidence of reliability and validity to measure the Oral Health Impact Profile OHIP-14 in the southern Peruvian population that speaks this language. These results will allow primary care dentists to have a useful, simple, and brief measurement tool to evaluate aspects related to oral health-related quality of life. Therefore, this culturally adapted instrument represents an important contribution to the assessment of indigenous and ethnic minority groups in Peru.

In conclusion, the OHIP-14 translated into Collao Quechua has acceptable properties and can be used as a tool for measuring quality of life indicators related to the oral health of the indigenous peoples of southern Peru who speak this language.

## Data Availability

The authors declare that all data generated or analyzed during this study are included in this published article.
